# Transcriptomic Profiling of Diverse *Aedes aegypti* Strains Reveals Increased Basal-level Immune Activation in Dengue Virus-refractory Populations and Identifies Novel Virus-vector Molecular Interactions

**DOI:** 10.1371/journal.pntd.0002295

**Published:** 2013-07-04

**Authors:** Shuzhen Sim, Natapong Jupatanakul, José L. Ramirez, Seokyoung Kang, Claudia M. Romero-Vivas, Hamish Mohammed, George Dimopoulos

**Affiliations:** 1 W. Harry Feinstone Department of Molecular Microbiology and Immunology, Bloomberg School of Public Health, Johns Hopkins University, Baltimore, Maryland, United States of America; 2 Laboratorio de Enfermedades Tropicales, Departamento de Medicina, Fundación Universidad del Norte, Barranquilla, Colombia; 3 Health Sciences, University of Trinidad and Tobago, O'Meara Campus, Arima, Trinidad and Tobago; National Institute of Allergy and Infectious Diseases, United States of America

## Abstract

Genetic variation among *Aedes aegypti* populations can greatly influence their vector competence for human pathogens such as the dengue virus (DENV). While intra-species transcriptome differences remain relatively unstudied when compared to coding sequence polymorphisms, they also affect numerous aspects of mosquito biology. Comparative molecular profiling of mosquito strain transcriptomes can therefore provide valuable insight into the regulation of vector competence. We established a panel of *A. aegypti* strains with varying levels of susceptibility to DENV, comprising both laboratory-maintained strains and field-derived colonies collected from geographically distinct dengue-endemic regions spanning South America, the Caribbean, and Southeast Asia. A comparative genome-wide gene expression microarray-based analysis revealed higher basal levels of numerous immunity-related gene transcripts in DENV-refractory mosquito strains than in susceptible strains, and RNA interference assays further showed different degrees of immune pathway contribution to refractoriness in different strains. By correlating transcript abundance patterns with DENV susceptibility across our panel, we also identified new candidate modulators of DENV infection in the mosquito, and we provide functional evidence for two potential DENV host factors and one potential restriction factor. Our comparative transcriptome dataset thus not only provides valuable information about immune gene regulation and usage in natural refractoriness of mosquito populations to dengue virus but also allows us to identify new molecular interactions between the virus and its mosquito vector.

## Author Summary

Genetic variations among *Aedes aegypti* mosquito populations can greatly influence their ability to transmit human pathogens such as the dengue virus (DENV). Some of these variations between mosquito populations are represented by differences in the expression of specific genes that control susceptibility to a pathogen. We have compared susceptibilities to dengue virus infection and the genome-wide gene expression patterns between laboratory-maintained *Aedes aegypti* strains as well as field-derived colonies collected from *geographically-distinct* dengue-endemic regions spanning South America, the Caribbean, and Southeast Asia. These analyses in conjunction with functional gene silencing assays showed that the basal immune activity is a likely determinant of resistance to the dengue virus, along with other novel factors. Our study also identified two potential DENV host factors and one potential restriction factor, thereby elucidating novel aspects of dengue virus – mosquito interactions.

## Introduction

With 3.6 billion people now living in areas at risk for epidemic transmission, dengue has become the most important mosquito-borne viral disease affecting humans [1]. Dengue fever (DF) and dengue hemorrhagic fever (DHF) are caused by four closely related but antigenically distinct dengue virus (DENV) serotypes (DENV1, 2, 3, and 4), all of which are transmitted primarily by the *Aedes aegypti* mosquito vector, and secondarily by *Aedes albopictus*. The incidence and geographic range of dengue has increased dramatically in recent decades, with hyperendemicity (epidemics caused by multiple serotypes) and DHF outbreaks becoming increasingly common [2], [3]. These trends can in large part be attributed to the success of the mosquito vectors of DENV, which are now well established in most tropical countries. Since there is at present no licensed vaccine or drug treatment against DENV, vector control remains the most effective method for curbing transmission.

Mosquitoes, like all insects, are exposed to a variety of microbes in their natural habitats and possess an innate immune system that is capable of mounting a potent response against microbial challenge. However, studies of mosquito immune responses to DENV and other human pathogens have largely been performed on laboratory strains of *A. aegypti*, which have been maintained under insectary conditions for many generations (i.e., decades). As compared to natural mosquito populations, laboratory mosquito strains are exposed to lower doses and a much narrower range of microbes; together with the genetic bottleneck of a small initial parental population size, this often results in a loss of genetic variability [4]. In contrast, sequence diversity of immunity-related genes is predicted to be higher in field mosquitoes, to allow them to cope with the broad range of microbes with which they come into contact [5]. Immunity-related gene polymorphisms can also be expected among natural mosquito populations from geographically distinct regions, since they would have co-evolved with distinct arbovirus genotypes as well as varying suites of microbial species.

These differences may have important consequences for vector-pathogen interactions and therefore for vector competence: for example, intraspecific sequence polymorphisms in immune genes have been correlated with the prevalence of *Plasmodium falciparum* infections in *Anopheles gambiae* field populations [6], as well as with refractoriness to the parasite [7]. There is also ample evidence that *A. aegypti* populations from different geographical locations vary in vector competence for arboviruses, including the four DENV serotypes [8]–[11], although this variation has not been specifically correlated with polymorphisms of immune genes.

Although most studies of genetic variation in mosquitoes have focused on coding sequence polymorphisms, *A. aegypti* populations are also likely to differ at the transcriptome level, either in the magnitude of the gene regulation or in the subsets of genes regulated. Importantly, gene expression is thought to evolve by at least an order of magnitude faster than gene sequences themselves [12], [13], probably because expression can be affected by both *cis-* and *trans*-acting transcription factors or DNA elements, and so the abundance of any one transcript can be affected by mutations in many different loci. Furthermore, a mutation in a regulatory locus can change the expression of numerous target genes (reviewed in [14]). Thus, comparative transcriptomics has great potential to reveal critical differences among strains that affect multiple aspects of mosquito physiology [15], [16].

In the present study, we have performed a comparative genome-wide microarray-based transcript abundance analysis on a panel of *A. aegypti* strains with varying levels of susceptibility to DENV, comprising both laboratory-maintained strains and field-derived colonies collected from geographically distinct dengue-endemic regions spanning South America, the Caribbean, and Southeast Asia. Our analysis revealed intriguing patterns of differential immune transcript abundance which, together with functional evidence from RNA interference assays, suggested that differences in basal levels of immune signaling among mosquito strains is an important factor in determining susceptibility to DENV. In addition, our dataset and panel of mosquito strains served as a valuable platform for the identification and functional characterization of candidate new modulators of DENV in *A. aegypti*, thus helping to expand our limited knowledge of DENV-mosquito molecular interactions.

## Materials and Methods

### Ethics statement

This study was carried out in strict accordance with the recommendations in the Guide for the Care and Use of Laboratory Animals of the National Institutes of Health. Mice were only used for mosquito rearing as a blood source according to approved protocol. The protocol was approved by the Animal Care and Use Committee of the Johns Hopkins University (Permit Number: M006H300). Commercial anonymous human blood was used for dengue virus infection assays in mosquitoes, and informed consent was therefore not applicable. The Johns Hopkins School of Public Health Ethics Committee has approved this protocol. Mosquito collections were performed in residences after owners/residents permission.

### Field collection and establishment of laboratory colonies of*A. aegypti*

Field-derived mosquito strains were collected as larvae from breeding sites (mostly domestic dwellings) in the field locations, with the exception of the Saint Kitts strain, which was collected as eggs laid on oviposition traps. Global positioning system (GPS) coordinates of the field collection sites are listed in Table 1. Larvae were raised to adulthood under insectary conditions at the field location, confirmed by visual inspection through a dissecting stereoscope as *A. aegypti*, and bloodfed to produce eggs (F0 generation). F0 eggs were brought back to the Johns Hopkins insectary, where they were hatched and again raised to adulthood, mated and bloodfed to produce F1 eggs. F1 individuals were used in the microarray analysis and to establish field-derived colonies in our insectary.

**Table 1:** Origins and name abbreviations of laboratory and field-derived*A. aegypti* strains.

Strain	Abbreviation	Lab/Field	Origin/Approx. date of colonization	GPS coordinates of collection site	Reference
Rockfeller	Rock	Lab	Caribbean/∼1930s	Unknown	[74]
Orlando	Orl	Lab	Orlando, Florida/∼1940s	Unknown	[75]
Waco	Waco	Lab	Waco, Texas/∼1987	Unknown	[76]
Puerto Rico	PR	Field	Carolina, Puerto Rico/2010	18°27′6″N, 66°4′8″W	-
Saint Kitts	Kitts	Field	Saint Kitts/2010	17°17′27″N, 62°41′27″W	-
Por Fin	PFin	Field	Por Fin, Barranquilla, Colombia/2010	10°58′23″N, 74°49′43″W	-
Puerto Triunfo	PTri	Field	Puerto Triunfo, Colombia/2010	5°52′0″N, 74°39′0″W	-
Singapore	SIN	Field	Singapore, Singapore/2010	1°22′12.6″N, 103°50′44.23″E	-
Bangkok	BKK	Field	Bangkok, Thailand/2011	13°39′12″N, 100°24′19″E	-

### Mosquito rearing and cell culture conditions

Mosquito strains were maintained on 10% sucrose solution at 27°C and 95% humidity with a 12 h light/dark cycle. The C6/36 *Aedes albopictus* cell line was maintained in MEM (Gibco) supplemented with 10% heat-inactivated FBS, 1% L-glutamine, 1% non-essential amino acids, and 1% penicillin-streptomycin. BHK-21 (clone 15) hamster kidney cells were maintained on DMEM (Gibco) supplemented with 10% FBS, 1% L-glutamine, 1% penicillin-streptomycin, and 5 ug/ml Plasmocin (Invivogen). C6/36 cells were incubated at 32°C and 5% CO_2_, and BHK-21 cells were incubated at 37°C and 5% CO_2_.

### Mosquito infections and assessment of vector competence for DENV2

Mosquito infections with DENV were carried out as previously described [17]. The New Guinea C strain of DENV2 (DENV2-NGC) was propagated in C6/36 cells: Cells seeded to 80% confluence in 75-cm^2^ flasks were infected with virus stock at a multiplicity of infection (MOI) of 3.5 and incubated for 6 days at 32°C and 5% CO_2_. Infected cells were scraped into solution and lysed to release virus particles by repeated freezing and thawing on dry ice and in a 37°C water bath. This yielded a virus titer of between 10^6^ and 10^7^ PFU/ml. Each virus suspension was mixed 1∶1 with commercial human blood and supplemented with 10% human serum and 10% 10 mM ATP. For experiments involving an uninfected control, a flask of uninfected C6/36 cells was maintained under similar conditions and used to create a naïve bloodmeal. The bloodmeal was maintained at 37°C for 30 min and then offered to mosquitoes via an artificial membrane feeding system. Infection assays done to assess vector competence were carried out with field-derived mosquitoes from the F5 generation or earlier. Midguts were dissected at 7 days post-blood meal (dpbm) and stored individually in DMEM at −80°C until titrated by plaque assay.

### Sample collection and processing for microarray-based transcript abundance analysis

Field-derived mosquitoes used for the microarray-based transcript abundance analyses were from the F1 generation. Larvae from each field or laboratory strain were reared at equal densities, and adults were maintained on 10% sucrose solution in cages of equal dimensions. Midguts and carcasses from 5-day-old adult female mosquitoes were dissected in RNAlater (Qiagen) and stored in RLT buffer (Qiagen) with 1% β-mercaptoethanol. Three independent biological replicates were collected, with at least 10 midguts or carcasses per replicate. Total RNA was extracted from samples using the RNeasy Mini kit (Qiagen).

### Microarray-based transcript abundance analysis

The Low Input Quick Amp Labeling kit (Agilent Technologies) was used to synthesize Cy-3- or Cy-5-labeled cRNA probes from 200 ng of total RNA per replicate. Probes from each strain were individually hybridized against probes from the Rockefeller laboratory strain as a control. Hybridizations were carried out on an Agilent-based microarray platform using custom-designed whole-genome 8 x 60K *A. aegypti* microarrays, and arrays were scanned with an Agilent Scanner. Transcript abundance data were processed and analyzed as previously described [18]–[20]; in brief, background-subtracted median fluorescent values were normalized with the LOWESS normalization method, and Cy5/Cy3 ratios from replicate assays were subjected to t-tests at a significance level of p<0.05 using TIGR MIDAS and MeV software. Transcript abundance data from replicate assays were averaged with the GEPAS microarray preprocessing software (http://www.babelomics.org) and logarithm (base 2)-transformed. Self-self hybridizations have been used to determine a cutoff value for the significance of gene regulation on these microarrays of 0.78 in log_2_ scale, which corresponds to 1.71-fold regulation [21]. Numeric microarray gene expression data are presented in Table S2.

Hierarchical (complete linkage) clustering was performed using Cluster, and outputs were visualized using Treeview (http://rana.lbl.gov/EisenSoftware.htm) [22]. Gene ontology (GO) class over-representation analysis was performed with the GO-Elite program (http://www.genmapp.org/go_elite) using the permute p-value algorithm; results were pruned to include only GO classes with at least three regulated genes, Z-score>1.96, and p-value<0.05. Numerical data from this analysis are presented in Table S4.

### Gene silencing assays

RNA interference (RNAi)-mediated candidate gene silencing in mosquitoes was performed as previously described [18], [19], [23]. Field-derived mosquitoes used in these assays were from generations F5 to F13, depending on the strain; however, mosquitoes within each strain were from the same generation to ensure valid comparison between experimental and dsGFP-treated groups.

Three-day-old female mosquitoes were cold-anesthetized and individually injected with 200 ng of dsRNA specific for the target gene of interest. Mosquitoes injected with dsRNA to GFP were used as controls. At 3–4 days post-injection, mosquitoes were fed on DENV2-NGC-supplemented blood. Midguts were dissected at 7 dpbm and stored individually in DMEM at −80°C until they were titered by plaque assay. dsRNA was synthesized using the HiScribe T7 *in vitro* transcription kit (New England Biolabs). Primer sequences used for dsRNA synthesis and to confirm gene silencing by real-time PCR are presented in Table S6. Due to high sequence identity, it was unavoidable that dsRNA against AAEL010429 also targeted AAEL013577 and AAEL010436 (also putative insect allergen family members), and dsRNA against AAEL015337 also targeted AAEL010599 (also a neutral alpha-glucosidase ab precursor). We cannot however exclude the possibility that these seemingly different transcripts represent the same gene, due to possible genome sequence annotation errors.

While the MyD88, Cactus, Imd, Caspar, Dome, PIAS and Dcr2 genes have been routinely silenced in mosquitoes by us and others reaching silencing levels ranging from 15–70%, gene silencing validation of genes that have previously not been silenced in mosquitoes are presented in Figure S2.

### DENV titration by plaque assay

DENV2-NGC titers in midguts were determined by plaque assay on BHK-21 (clone 15) cells. Individual midguts were homogenized in DMEM with a Bullet Blender (NextAdvance), serially diluted, and then inoculated onto cells seeded to 80% confluence in 24-well plates (100 µl per well). Plates were rocked for 15 min at room temperature, and then incubated for 45 min at 37°C and 5% CO_2_. Subsequently, 1 ml of DMEM containing 2% FBS and 0.8% methylcellulose was added to each well, and plates were incubated for 5 days at 37°C and 5% CO_2_. Plates were fixed with a methanol/acetone mixture (1∶1 volume) for at least 1 h at 4°C, and plaque-forming units were visualized by staining with 1% crystal violet solution for 10 min at room temperature.

### Statistical analysis of DENV infection and gene silencing assays

At least three biological replicates of each DENV infection or gene silencing assay were performed, except for DENV4-WRAIR infections and the initial DENV2-NGC infections of PTri, BKK and PR, where two biological replicates were performed. Plaque assay data were pooled for each experimental group. The number of mosquitoes in each biological replicate did not vary greatly (Table S1), and infecting DENV titers did not vary by more than 1 log PFU/ml among replicates. Either the Kruskal-Wallis test with Dunn's post-test or the Mann-Whitney test was used to detect significant differences in DENV titers among experimental groups.

## Results

### Laboratory and field-derived strains of*A. aegypti* show varying degrees of susceptibility to DENV2-NGC midgut infection

We established a panel of eight laboratory- and field-derived *A. aegypti* strains (Table 1), obtained either through generous contributions from collaborators or our own field collections. Field locations were selected to represent geographically distinct dengue-endemic regions spanning South America, the Caribbean, and Southeast Asia, and laboratory strains were included to allow us to study the effect of long-term colonization on the transcriptome.

**Figure 1:**
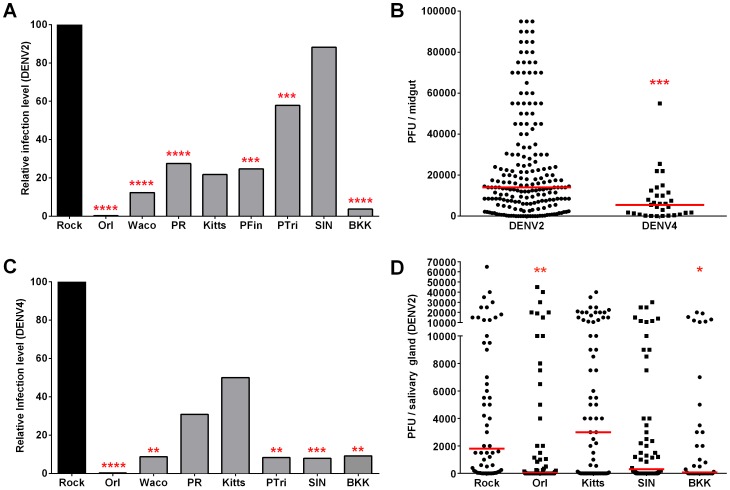
Susceptibilities of*A. aegypti* strains to DENV infection. (A) Relative median DENV2-NGC midgut infection levels at 7 days post-bloodmeal (dpbm) for each *A. aegypti* strain, compared to its respective Rock control. (B) Midgut DENV2-NGC and DENV4-WRAIR titers at 7 dpbm in the Rock strain. Each data point represents an individual midgut. (C) Relative median DENV4-WRAIR midgut infection levels at 7 dpbm for each *A. aegypti* strain, compared to its respective Rock control. (D) Salivary gland DENV2-NGC titers at 14 dpbm. Each data point represents an individual salivary gland. (A–D) Data are a pool of at least three independent biological replicates, except for DENV4-WRAIR infections and DENV2-NGC infections of PTri, BKK and PR, where two biological replicates were performed. ****, p≤0.0001; ***, p≤0.001; **, p≤0.01 *, p≤0.05 compared to Rock in Dunn's post-test after Kruskal-Wallis test or in Mann-Whitney test.

To assess their susceptibility for DENV, we orally infected each mosquito strain with the New Guinea C strain of DENV2 (DENV2-NGC) and assessed midgut virus titers at 7 dpbm. The highly susceptible Rockefeller (Rock) laboratory strain typically used in our group's experiments [18], [19], [24] served as a basis for comparison. Because the PR, PTri, and BKK strains were obtained at later times, it was necessary to perform separate Rock strain controls for each of these (Figure S1). To rank all strains in the panel by susceptibility to midgut infection, we calculated the relative infection level of each strain by taking its median DENV2-NGC titer as a percentage of the median titer of its respective Rock control (Figure 1A). Virus titers from individual mosquitoes are presented in Figure S1 and descriptive statistics in Table S1.

DENV2-NGC replicated to significantly lower midgut titers in Orl, Waco, PFin, PR, PTri, and BKK as compared to Rock, and median virus titers in Kitts were four-fold lower than in Rock (Figure 1A). SIN was the only strain in which DENV2-NGC replicated to titers comparable to Rock (Figure 1A). Compared to Rock, the Orl and BKK strains were the most refractory to DENV2-NGC midgut infection, while the SIN and PTri strains were the most susceptible (Figure 1A).

Next, to determine whether the susceptibility ranking we observed was specific to DENV2-NGC, we also assessed the midgut susceptibility of the mosquito strains to the WRAIR strain of DENV4 (DENV4-WRAIR). DENV4 was chosen because it is the most genetically divergent from DENV2 of all the serotypes [25].

DENV4-WRAIR replicated to much lower midgut titers than did DENV2-NGC across all mosquito strains in the panel (Figure S1E); data for Rock are shown as a representative example in Figure 1B. Compared to Rock, Orl and BKK were again among the most refractory in the panel, and, in contrast to what was observed for DENV2-NGC, PTri and SIN were also highly refractory (Figure 1C). Since absolute DENV4-WRAIR midgut titers were much lower across all strains, this refractoriness may be a result of threshold infection levels not having been reached, or alternatively may be due to specific differences in how PTri and SIN mosquito factors act on different DENV serotypes. This finding differs from a previous study in which the susceptibility patterns of a panel of field-derived *A. aegypti* were found to be similar across all four DENV serotypes [11].

Although establishing a midgut infection is crucial for productive infection of the mosquito, the virus encounters a second bottleneck when it reaches the salivary gland. Since midgut susceptibility may be an unreliable predictor of salivary gland infection, and given the importance of the gland for horizontal DENV transmission, we also measured salivary gland DENV2-NGC titers at 14 dpbm for the Rock, Orl, BKK, Kitts, and SIN strains (Figure 1D). We did not include all mosquito strains in this analysis because of the more tedious nature of salivary gland dissection. As in the midgut, Orl and BKK again displayed the lowest salivary gland titers (Figure 1D). In contrast to the midgut (Figure 1A, Figure S1), median SIN salivary gland titers were 6-fold lower than those of Rock, and median Kitts salivary gland titers were almost twice as high as Rock titers (Figure 1D). These data suggest that while mosquito strains that support very low midgut infection levels are also likely to show poor virus dissemination to the salivary gland, it is difficult to predict downstream virus replication kinetics in strains that support moderate-to-high midgut infection levels.

### Comparative microarray-based analysis of*A. aegypti* basal-level transcriptomes

To characterize and identify transcriptomic variations that could affect susceptibility to DENV or other aspects of mosquito physiology, we used whole-genome oligonucleotide microarrays to compare basal (non-DENV-infected) transcript abundances in the midguts and carcasses of each strain in the panel to our standard Rock strain. Not surprisingly, for each strain, hundreds of transcripts from a range of functional classes were significantly differentially represented when compared to Rock, ranging from 376 in the midgut of the SIN strain to 715 in the carcass of the BKK strain (Table S3).

**Figure 2:**
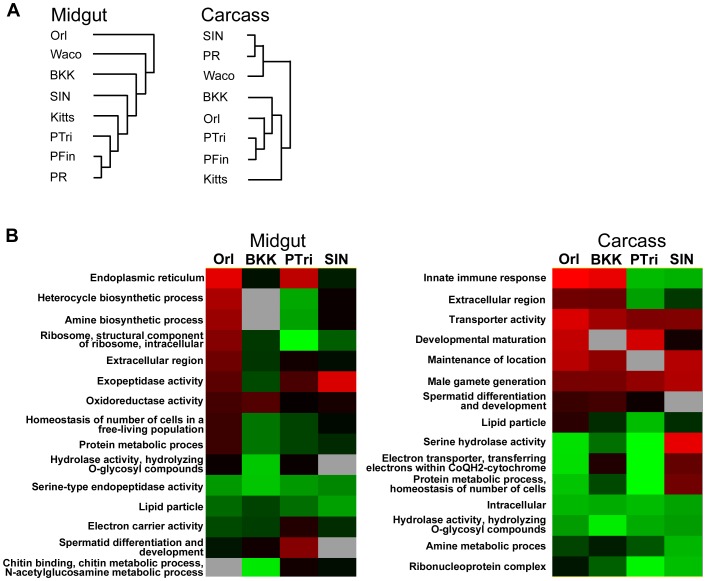
Comparative microarray-based profiling of the basal-level transcriptomes of laboratory and field-derived*A. aegypti* strains. (A) Hierarchical clustering of transcript abundance profiles in the midgut and carcass. Genes that displayed significant differential transcript abundance in at least four of the eight mosquito strains (compared to the Rock strain) were included in the cluster analysis. (B) Over-represented Gene Ontology (GO) classes in the set of genes that displayed significant differential transcript abundance in the most refractory and most susceptible *A. aegypti* strains. Red and green indicate average log_2_-fold higher and lower transcript abundance, respectively, for each class. The criteria for over-representation were: at least 3 genes displaying differential transcript abundance per class, z-score >1.96, and p value <0.05. MG, midgut; Car, carcass.

To obtain a global picture of the physiological relatedness of our mosquito strains, we performed hierarchical clustering [22] on their transcriptomes. In the midgut, transcript abundance profiles of the field-derived strains were more closely correlated with one another than with the Orl and Waco laboratory strains (Figure 2A). In addition, the transcriptome-relatedness of the field-derived strains correlated roughly with their geographical origin, with the Caribbean and South American strains being more closely related to one another than to the two Southeast Asian strains (Figure 2A).

The clustering pattern was less clear in the carcass, with no distinction between laboratory and field-derived strain transcriptomes (Figure 2A). The transcriptomes of the two Colombian strains were highly correlated, as were the transcriptomes of the PR and SIN strains.

Notably, at this global transcript level, mosquito strains did not appear to cluster by susceptibility, suggesting that only a small subset of genes regulates vector competence through their transcript abundance.

### Transcripts of numerous immunity-related genes are enriched in refractory*A. aegypti* strains

We were particularly interested in the differential expression patterns of immunity-related transcripts. In the carcass, the refractory Orl and BKK strains displayed the highest numbers of significantly more abundant immune transcripts in the panel, compared to Rock (23 and 31 respectively), while the susceptible PTri and SIN strains displayed among the lowest (10 and 8) (Table S3).

Both refractory and susceptible strains displayed immune transcripts that were significantly less abundant than in Rock (Table S3). This was more apparent in the midgut than in the carcass - Orl and BKK midguts displayed 10 and 25 significantly less abundant immune transcripts respectively. Despite this, Orl and BKK midguts still displayed higher numbers of significantly enriched immune transcripts compared to PTri and SIN (5 and 12 compared to 2 and 1) (Table S3).

Strikingly, numerous transcripts with known or putative immune-related functions were significantly enriched in one or both of the refractory Orl and BKK strains but not in any other strain, suggesting that they may contribute to refractoriness or play unique roles in these strains.

Five immune-related transcripts were uniquely more highly abundant in the carcass of the Orl strain, including three cecropins (AAEL000611, AAEL015515, and AAEL000621; 4.71-, 4.08-, and 2.78-fold enriched in log_2_ scale respectively), one galactose-specific C-type lectin (CTL) (AAEL014382; 1.45 log_2_-fold), and a clip domain serine protease (AAEL005431; 0.97 log_2_-fold). Our previous transcriptome studies [18] indicated that these transcripts were also induced upon activation of the Toll pathway. In addition, transcripts of a putative pupal cuticle protein (AAEL004758) were uniquely and strongly enriched in both the midgut and carcass of the Orl strain (5.17 and 2.13 log_2_-fold respectively). A recent study identified a pupal cuticle protein that, when over-expressed in adult mosquitoes, results in reduced West Nile virus (WNV) titers [26]. Since this protein was able to bind both WNV and DENV, the authors hypothesized that it may sequester virions and prevent them from infecting cells [26].

Transcripts of sixteen immune-related genes were uniquely enriched in the midgut or carcass of the BKK strain. These included four cathepsin b (AAEL007585, AAEL012216, AAEL015312, and AAEL009642; 2.98, 2.88, 2.66, and 2.60 log_2_-fold) genes, three fibrinogen-related protein (AAEL013417, AAEL000726, and AAEL008646; 0.81, 1.06, and 0.99 log_2_-fold) genes, two prophenoloxidase (AAEL013498 and AAEL015116; 0.81 and 0.79 log_2_-fold) genes, and the Tep2 (AAEL014755; 0.78 log_2_-fold) gene.

Strikingly, only transcripts of a leucine-rich repeat-containing transmembrane protein (AAEL005762; 0.79 log_2_-fold) were uniquely more abundant in the midgut of the PTri strain, and only transcripts of a peroxinectin (AAEL004401; 0.93 log_2_-fold) gene were uniquely more abundant in the carcass of the SIN strain.

We next considered Gene Ontology (GO) classes instead of individual transcripts. Using the GO-Elite program (http://www.genmapp.org/go_elite), we determined that the “innate immune response” and “extracellular region” GO classes were over-represented among the differentially abundant transcripts in the carcasses of the Orl, BKK (refractory), PTri, and SIN (susceptible) strains (Figure 2B). In addition to being over-represented, these GO classes also comprised transcripts that, on average, were enriched in the refractory strains and depleted in the susceptible strains (Figure 2B). The “innate immune response” class included several antimicrobial peptides (AMPs), which in addition to their antibacterial properties have also been shown to have anti-DENV activity [27], [28]. The “extracellular region“ class refers to gene products that are secreted and that are not attached to the cell surface, and here they included lysozymes, transferrins, insect allergens, and a peroxinectin, all of which have known or potential roles in insect immunity [19], [29]–[31]. These GO classes were not over-represented in the midguts of these strains (Figure 2B).

In summary, basal abundance levels of numerous immunity-related transcripts were higher in the refractory strains in our study than in susceptible strains, suggesting that differences in baseline immune system activation may contribute towards determining vector competence.

### Mosquito immune signaling pathways and the RNAi pathway control DENV2-NGC infection to different degrees in various*A. aegypti* strains

Three major immune signaling pathways, the Toll, IMD, and JAK-STAT pathways, direct the mosquito immune response to a variety of pathogens (reviewed in [32]). Since these pathways are likely to regulate many of the immunity-related transcripts found to be enriched in the Orl and BKK strains, we next examined the contributions of these pathways to refractoriness in these strains. In addition, since the RNAi pathway is an important controller of DENV infection in *A. aegypti* [33], we also examined its role in this regard. Each pathway was compromised through the RNAi-mediated knockdown of a key pathway component, and the effect of this manipulation on midgut DENV2-NGC titers was assessed.

**Figure 3:**
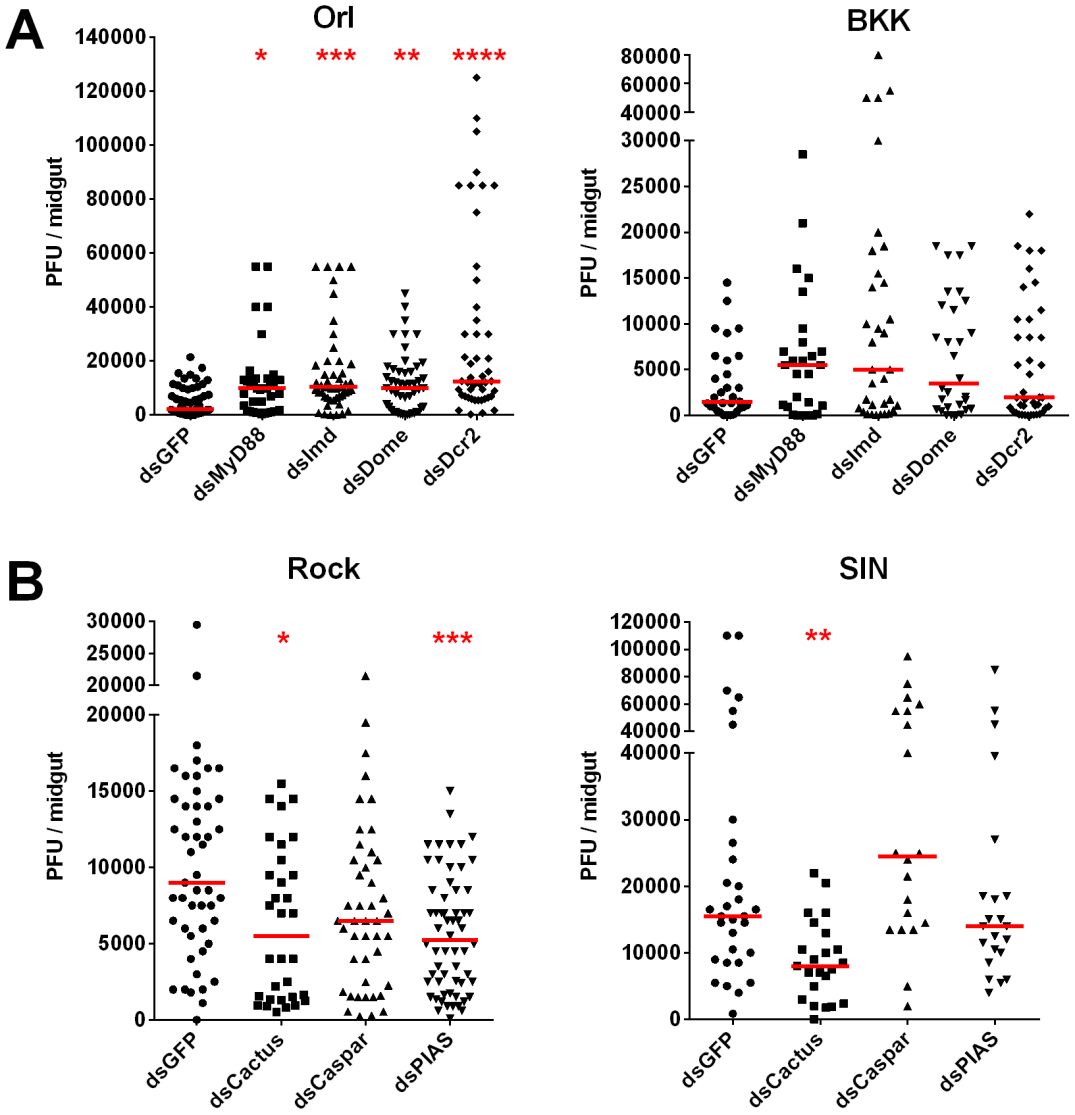
Contributions of the Toll, IMD, JAK-STAT, and RNAi pathways to the control of DENV2-NGC in refractory and susceptible mosquito strains. Midgut DENV2-NGC titers at 7 dpbm in (A) MyD88, IMD, Dome, and Dcr2-silenced Orl and BKK mosquitoes, and (B) Cactus, Caspar, and PIAS-silenced Rock and SIN mosquitoes. Data are a pool of at least three independent biological replicates. ****, p≤0.0001; ***, p≤0.001; **, p≤0.01 *, p≤0.05 compared to dsGFP-treated mosquitoes in Dunn's post-test after Kruskal-Wallis test.

In the Orl strain, individually compromising the Toll, IMD, JAK-STAT, and RNAi pathways via knockdown of MyD88, IMD, Dome, and Dcr2, respectively, resulted in a significant and dramatic increase in midgut virus load (Figure 3A), to titers comparable to those typically seen in Rock (Figure S1A–D), suggesting that each of these pathways is a major contributor to the refractoriness seen in Orl. In the BKK strain, compromising the Toll, IMD, JAK-STAT or RNAi pathways only resulted in a non-significant 3- to 5-fold increase in midgut virus titers (Figure 3A), suggesting the action of BKK strain-specific DENV restriction factor(s) that operate independently of these pathways (Figure 3A).

Conversely, activating immune signaling pathways by silencing pathway negative regulators should render the susceptible Rock and SIN strains more refractory. In agreement with our previous studies [18], [19], silencing the Toll and JAK-STAT pathway negative regulators Cactus and PIAS in the Rock strain resulted in a significant decrease in midgut DENV titers, while silencing the IMD pathway negative regulator Caspar resulted in a non-significant decrease (Figure 3B). As expected, silencing Cactus in the SIN strain also resulted in a significant decrease in virus titers, but no effect was seen for PIAS or Caspar. It is possible that the JAK-STAT and IMD pathways are already operating at maximum capacity, especially given the high viral load observed in this strain, or that the SIN strain possesses factors acting independently of these pathways that facilitate DENV infection.

In summary, these data suggest that while the major immune signaling pathways play a key role in determining DENV susceptibility in both laboratory- and field-derived mosquitoes, strain-specific factors acting independently of these pathways are also likely to make important contributions. We speculate that the basal activation levels of immune pathways may be higher in refractory mosquitoes, and that these elevated levels may contribute to refractoriness by increasing the transcript abundance of various immune effectors, as observed in our microarray analysis. It is also possible that immune pathways are more strongly activated in refractory strains in response to the recognition of invading pathogens such as DENV, although our microarray analysis did not address this possibility.

### Identifying candidate DENV host and restriction factors through hierarchical clustering

In addition to our interest in immunity-related genes, we also wanted to identify DENV host and restriction factors with previously unknown roles in modulating DENV replication. DENV host factors are genes whose products facilitate DENV infection or replication processes in the mosquito, while DENV restriction factors encode products that inhibit these processes in some fashion. For this purpose we conducted hierarchical cluster analyses of the transcriptomes of the two most refractory (Orl, BKK) and two most susceptible (PTri, SIN) mosquito strains. In addition to expanding our limited knowledge of molecular interactions between DENV and mosquito cellular factors, this analysis also offered the potential to identify molecular determinants that affect vector competence in field mosquitoes.

**Figure 4:**
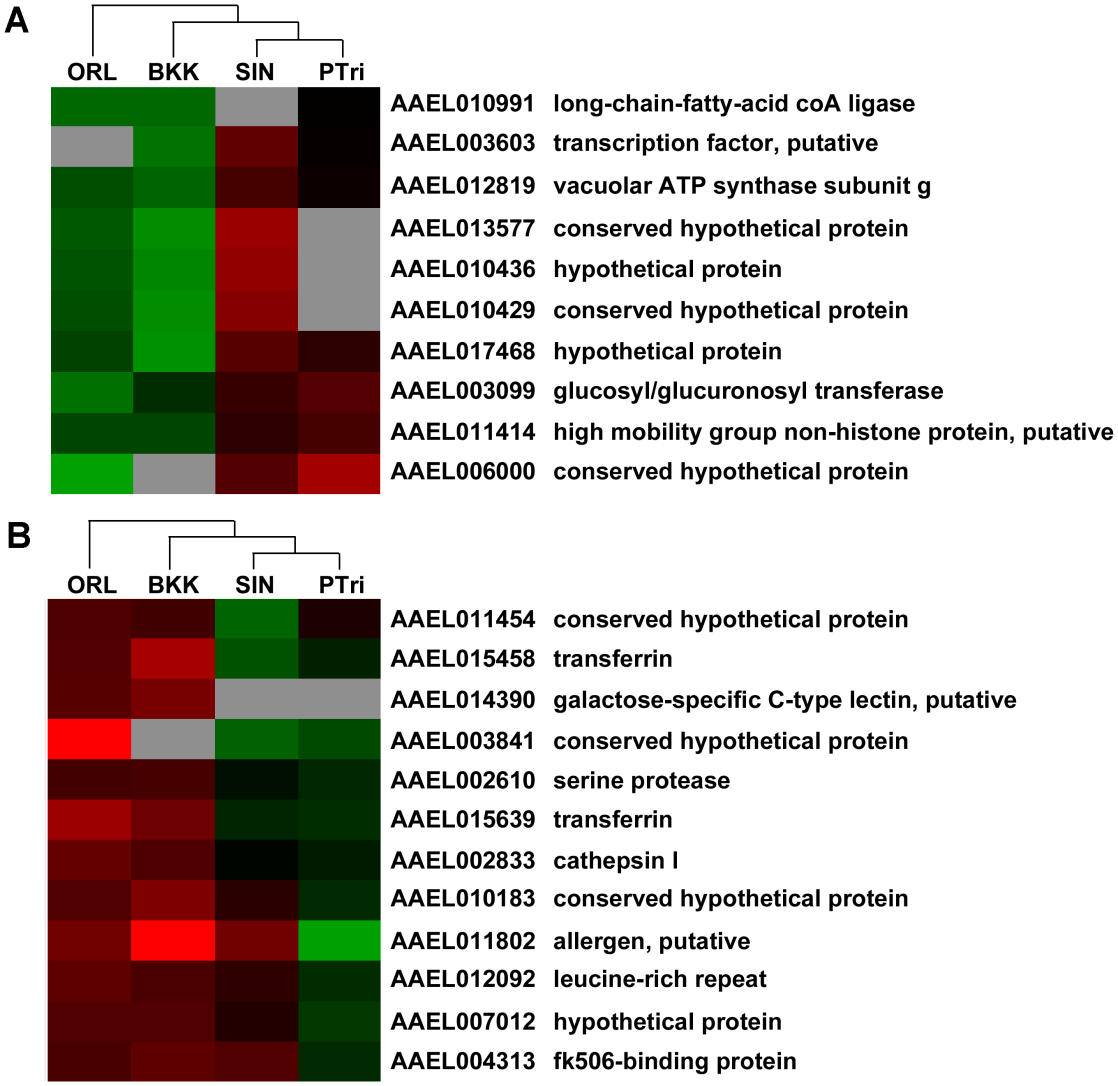
Identification of novel candidate DENV host and restriction factors through hierarchical clustering. Selected gene clusters obtained through hierarchical clustering of the midgut (A) and carcass (B) transcriptomes of the two most refractory (Orl, BKK) and two most susceptible (PTri, SIN) *A. aegypti* strains. Genes that displayed differentially abundant transcripts in at least two of the four strains were included in the analysis. Red and green indicate increased and decreased transcript abundance, respectively, compared to the Rock strain.

In the midgut, we identified a gene cluster that displayed an overall lower transcript abundance in the refractory Orl and BKK strains and a higher transcript abundance in the susceptible PTri and SIN strains (Figure 4A). We consider these genes to encode candidate DENV host factors. The cluster included a vacuolar ATPase (vATPase) subunit G (AAEL012819), a glucosyl/gluronosyl transferase (AAEL003099), a putative high mobility group non-histone protein (AAEL011414), and three hypothetical protein genes that encoded insect allergen repeats (AAEL013577, AAEL010436, and AAEL010429).

vATPases are multisubunit enzymes found in the membranes of endosomes, lysosomes, and secretory vesicles, where they play important roles in the acidification of these organelles [34]. Acidification of late endosomes is required for DENV fusion and entry into host cells [35]; indeed, bafilomycin, a specific inhibitor of vATPases, is a well-known flavivirus inhibitor [36], [37]. There is also evidence that DENV prM binds vATPase and that vATPase activity is also required for DENV egress [38].

Glucosyl/gluronosyl transferases catalyze the transfer of glycosyl groups from nucleotide sugars to xenobiotic compounds. This process renders xenobiotics more hydrophilic and facilitates their subsequent metabolism and excretion [39]. Since glycosylation of the DENV NS1 protein is required for its stability and secretion [40], it is possible that DENV may hijack this pathway; however, there is currently no evidence for this activity.

The putative high-mobility group non-histone protein AAEL011414 is a member of the high-mobility group box (HMGB) protein family. HMGB proteins bind DNA and are abundant in the nucleus, where they regulate chromatin structure and transcription. However, they are also found in the cytoplasm and extracellularly, where they trigger pro-inflammatory responses, tissue repair responses, and immune cell activation in mammalian systems [41]. HMGB1 released from DENV-infected human dendritic cells stimulates inflammatory cytokine production from co-cultured T cells, resulting in control of virus replication [42]. More recently, HMGB proteins have been found to bind immunogenic cytosolic nucleic acids such as ssRNA and dsRNA and to be required for these nucleic acids to activate Toll-like receptor (TLR)-stimulated immune responses [43]. The transcript abundance pattern of AAEL011414 observed here, while striking, does not fit the model of the apparent immune-stimulatory role of HMGB proteins; however, these proteins may play different roles in mosquitoes.

The insect allergen repeat domain is widespread in insect species. It is found in cockroach allergens, which are a common cause of asthma [44], and in nitrile-specific detoxifier proteins in butterflies, which allow caterpillars to feed on certain plant species [45]. The function of these proteins in mosquitoes has not been studied. In addition to the intriguing transcript abundance pattern observed here, previous *A. aegypti* microarray-based transcriptome studies from our laboratory indicated that transcripts of five members of this family (including AAEL010429 and AAEL010436) were enriched upon activation of the JAK-STAT pathway, suggesting that they are regulated via this pathway [19].

Conversely, in the carcass, we identified a gene cluster whose transcripts were enriched in the Orl and BKK strains and either depleted or weakly enriched in the PTri and SIN strains (Figure 4B); these we consider to be candidate DENV restriction factors. This cluster included two transferrin (AAEL015458 and AAEL015639) genes, a cathepsin l (AAEL002833) gene, and a leucine-rich repeat (LRR) containing protein (AAEL012092) gene.

Transferrins bind iron with high affinity and play roles in iron metabolism, immunity, and development. They are up-regulated upon parasite or bacterial infection and may sequester iron from pathogens; alternatively, proteolytic fragments from these proteins have also been suggested to also act as anti-microbial peptides or inducers of the immune response [30]. In addition to the carcass, AAEL015458 transcripts were also enriched in the midgut of the Orl and BKK strains, but not in any of the other strains.

Cathepsin l is a lysosomal cysteine protease that plays important roles in immune activation in mammals (reviewed in [46]). For example, it is required to cleave TLRs 7 and 9 in the endolysosome before these molecules can signal [47]–[49], and it also enhances the activity of the human cytokine IL-8 via N-terminal truncation [50].

The broader LRR-containing protein family includes the mosquito Tolls and family members that are commonly involved in protein-protein interactions and signal transduction pathways [51]. AAEL012092 transcripts were also enriched upon Toll pathway activation [18], and the gene has no known paralogs in *A. aegypti*, suggesting a unique function for its protein product. In addition to the carcass, AAEL012092 transcripts were also enriched in the midguts of the Orl and BKK strains, but not in any of the other strains.

### Functional characterization of selected candidate DENV host factors

We selected three candidate DENV host factors (Table 2, Table S5) identified through hierarchical clustering for functional analysis in the susceptible Rock, PTri, and SIN mosquito strains and hypothesized that knockdown of these genes via RNAi-mediated gene silencing would render mosquitoes more refractory to DENV.

**Table 2:** Candidate DENV host factors selected for functional characterization via RNAi-mediated gene knockdown.

Gene ID	Name	Abbreviation	Functional group	Reference
AAEL011414	high-mobility group non-histone protein, putative	HMGB	MET	This study
AAEL010429, AAEL013577, AAEL010436	conserved hypothetical protein	Aller	DIV	This study
AAEL012819	vacuolar ATP synthase subunit G	vATP-G	TRP	This study
AAEL002579	hypothetical protein	HP2579	UNK	[37]
AAEL002430	N-acetylglucosamine-6-phosphate deacetylase	N-Gluc	MET	[37]
AAEL015337, AAEL010599	neutral alpha-glucosidase ab precursor (glucosidase ii alpha subunit) (alpha glucosidase 2)	a-Gluc	MET	[37]

Functional group abbreviations: MET, metabolism; DIV, diverse; TRP, transport; UNK, unknown.

**Figure 5:**
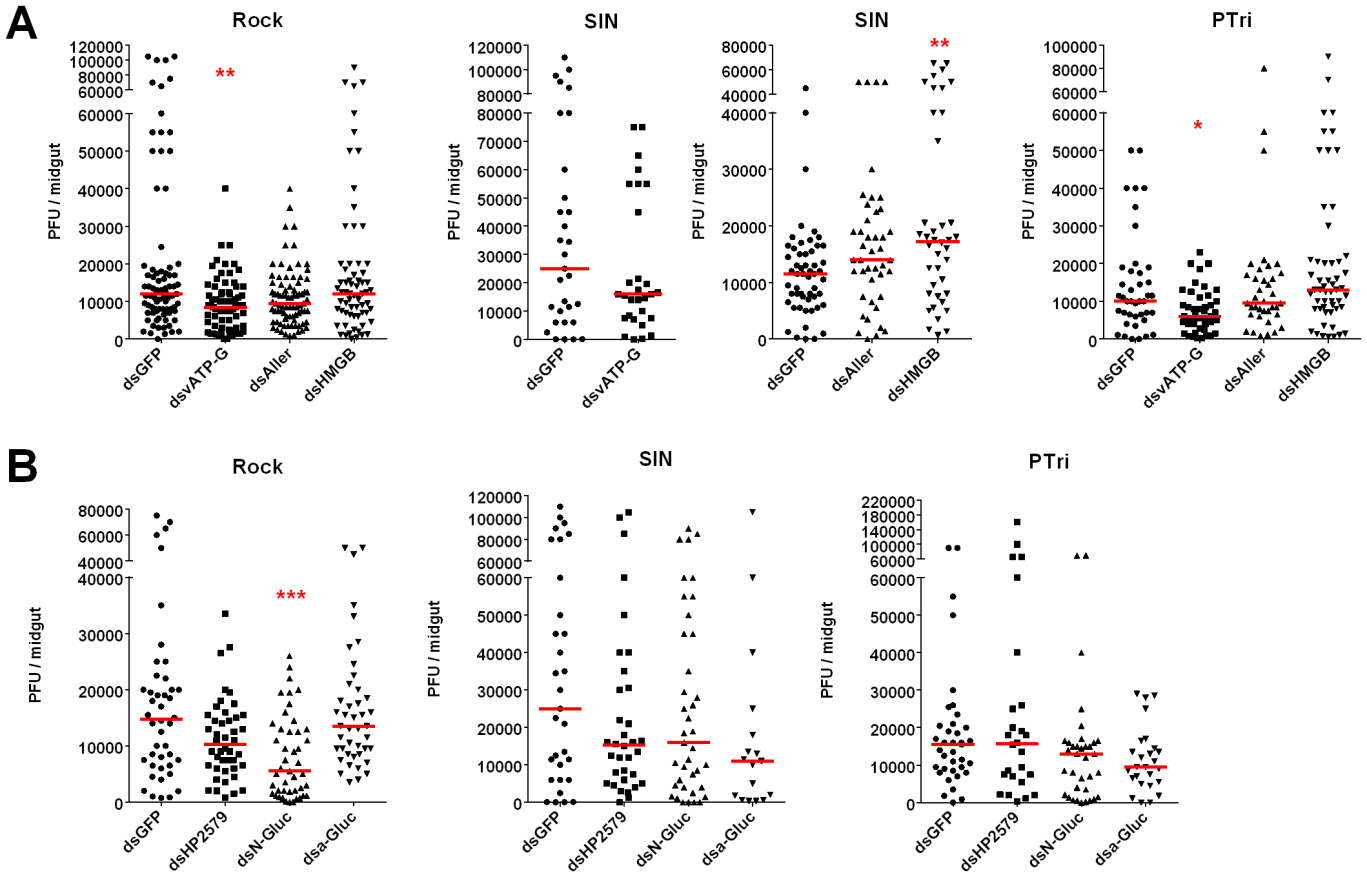
Effect of candidate DENV host factor knockdown on midgut DENV2-NGC titers in susceptible*A. aegypti* strains. Midgut DENV2-NGC titers at 7 dpbm in (A) vATP-G, Aller, and HMGB-silenced mosquitoes, and (B) HP2579, N-Gluc and a-Gluc-silenced mosquitoes. Data are a pool of at least three independent biological replicates. **, p≤0.01 *, p≤0.05 compared to dsGFP-treated mosquitoes in Dunn's post-test after Kruskal-Wallis test.

Knockdown of the vATPase subunit G gene (AAEL012819) resulted in significantly reduced midgut DENV titers in both the Rock and PTri strains and a non-significant decrease in the SIN strain, suggesting that it does indeed function as a DENV host factor (Figure 5A). Surprisingly, knockdown of the gene encoding a HMGB protein (AAEL011414) resulted in a significant increase in SIN strain DENV titers, but it had no effect in the other two mosquito strains (Figure 5A). Knockdown of the putative insect allergen had no effect in any of the strains.

Our panel of *A. aegypti* strains is also an excellent platform for characterizing or validating candidate DENV host factors identified through other screening methodologies. Sessions *et al.* (2009) identified numerous candidate DENV host factors by performing a high-throughput RNAi screen in *Drosophila* cells [37]. We selected the *A. aegypti* orthologs of three hits (Table 2, Table S5) from this study for functional characterization in our susceptible mosquito strains (Figure 5B). Only the knockdown of AAEL002430, which codes for an N-acetylglucosamine-6-phosphate deacetylase, resulted in significantly decreased DENV titers in the Rock strain but not in the SIN or PTri strains.

In addition to host factors, we also attempted to functionally characterize several candidate DENV restriction factors (Table S5) by silencing them in the Orl and BKK refractory mosquito strains. However, none of these factors were found to have an effect on midgut virus titers (data not shown). Since, as we have shown above, refractory strains are enriched in numerous and varied immunity-related transcripts, the depletion of a single restriction factor may only have a small impact on the insect's overall ability to defend against DENV. In contrast, host factors tend to perform unique functions in the mosquito cell, and thus host factor-DENV molecular interactions are less likely to be redundant.

## Discussion

While most transcriptomic variation may be neutral, numerous studies have shown that environmental factors such as geographic location, passage history, nutrient limitations, sex-dependent selection, and exposure to pathogens can exert effects on the transcriptome [5], [52]–[55], for example through the selection of mutations in *cis*- and *trans*-elements that regulate transcription [12], [56], [ reviewed in 57]. Although all the *A. aegypti* strains used in this study were raised under identical insectary conditions, their previous long-term exposure to the unique combination of environmental factors in their locations of origin is likely to have shaped their transcriptomes. For example, the role of natural midgut microflora in stimulating basal immunity in mosquitoes has been well described [18], [56], [58], and we speculate that the co-evolution of each strain with unique suites of microbial species is likely to have played a role in driving the transcriptomic divergence of immune genes. It is also possible that exposure to different circulating DENV serotypes and genotypes has shaped the DENV-responsive immune repertoires of these mosquito populations.

To examine the impact of transcriptomic variation on vector competence for DENV, we characterized and compared the basal midgut and carcass transcriptomes of a panel of laboratory and field-derived *A. aegypti* strains that differ in susceptibility to DENV2-NGC. While previous studies have also compared transcriptional profiles of refractory and susceptible *A. aegypti* strains [15], [16], our panel of strains is the largest, most diverse, and least passaged, and we are the first to attempt to functionally validate the role of differentially expressed genes in modulating vector competence.

Hierarchical clustering showed that midgut transcriptomic profiles of field-derived mosquito strains were more closely correlated to one another than to laboratory strains, and also clustered roughly by geographical origin. This may reflect midgut exposure to different suites of microbiota according to geographical location, although it is highly likely that microbial composition varies greatly even between microclimates in a single location. Other differences in midgut exposure may include nectar and blood sources. No clear pattern was observed among the carcass transcriptomic profiles - compared to the midgut, the carcass may be relatively more affected by environmental factors such as fluctuations in temperature and humidity, and contact with chemicals such as insecticides and biotoxins, making the effect on carcass transcriptomes difficult to predict.

A major finding of our study was the enrichment of numerous immunity-related transcripts in refractory, but not in susceptible mosquito strains. This finding, together with the ability of the Toll, JAK-STAT, and IMD pathways to control DENV infection in refractory strains, indicates that basal levels of mosquito immunity influence vector competence, in addition to (or instead of) other factors, such as the availability of receptors or host factors required for virus infection and replication. It further implies that any environmental factor that drives immune gene sequence or transcriptomic divergence has the potential to affect vector competence. While these may seem to be obvious conclusions, determinants of vector competence in natural mosquito populations are very poorly studied, and the idea of a basal level of immunity is not necessarily a given.

While observed in both compartments, basal enrichment of immune transcripts in refractory strains was much more apparent in the carcass than in the midgut. Many immune transcripts were in fact significantly less abundant in midguts of refractory strains. This is difference is intriguing and again suggests that the compartments experience distinct environmental exposures. The midgut also harbors non-pathogenic intestinal microflora that are likely to play important roles in nutrition or defense against insect pathogens [59]; thus basal transcript levels of immune effectors in this compartment are likely to be very tightly regulated in order to maintain homeostasis.

Our data also suggest that the importance of classical immunity varies among strains, with strain-specific non-immune DENV host or restriction factors exerting a greater influence in some mosquito populations.

Our transcriptomic dataset and panel of strains also allowed us to functionally characterize several candidate DENV host factors, which were selected because they displayed increased transcript abundance in susceptible strains but decreased transcript abundance in refractory strains. RNAi-mediated knockdown of the vATPase subunit G gene (vATP-G, AAEL012819) rendered susceptible mosquito strains more refractory to midgut DENV2-NGC infection, suggesting that it does indeed function as a DENV host factor. vATPases are multisubunit enzymes found in the membranes of endosomes, lysosomes, and secretory vesicles that bring about the acidification of these organelles via an ATP-dependent rotary mechanism that drives proton transport [34].This is an important step in the DENV replication cycle, since an acidic pH in the late endosome is required for DENV fusion and entry [35]. Although bafilomycin, a specific inhibitor of vATPases, has been reported to inhibit flaviviruses in both mammalian and insect cells [36], [37], this is to our knowledge the first functional evidence in adult *A. aegypti* for the role of the vATPase complex as a DENV host factor, and it suggests that this class of enzyme could be a promising target for chemical interventions, such as the development of small-molecule inhibitors of DENV replication.

In the vATPase complex, subunit G is part of a peripheral stalk connecting the peripheral domain (which catalyzes ATP hydrolysis) and the integral domain (through which protons are translocated), and it may play the role of stator in the rotary machinery. In yeast, deletion of subunit G leads to complete loss of assembly of the complex (as is true for all of the subunit genes except subunit H, whose deletion results in an inactive vATPase) [34]. In our dataset, vATP-G was the only subunit to show a clear pattern of enriched basal-level transcript abundance in susceptible strains. However, a recent study reported the enrichment of numerous vATPase subunit transcripts (including vATP-G) upon DENV infection of a susceptible African *A. aegypti* strain [15], suggesting multiple levels of transcriptional regulation for vATPase components. We are currently studying the effect of RNAi-mediated depletion of other key vATPase subunits on DENV replication in adult mosquitoes.

The knockdown of another candidate DENV host factor, a gene encoding a HMGB protein (AAEL011414), unexpectedly resulted in a significant increase in SIN midgut DENV titers, suggesting that it may function as a restriction factor instead in this strain. This is in agreement with the relatively well-studied role of this gene family in mammalian cells, in which human HMGB1 translocates out of the nucleus and is released from DENV-infected epithelial and dendritic cells, triggering a pro-inflammatory antiviral response [42], [60]. Serum HMGB1 levels are also elevated in DENV-infected patients [61]. While our functional data suggest that *A. aegypti* HMGB may play a similar antiviral role at least in the SIN strain, the transcript abundance pattern of this gene across our panel runs counter to this possibility. Since HMGB family members are also abundant in the nucleus, where they regulate chromatin structure, transcription, and DNA repair and replication [62], the transcription pattern we observed may have more to do with one or more of these functions than with a response to DENV. A recent study characterizing a separate *A. aegypti* HMGB family member (AAEL011380) confirmed that it, like human HMGB1, effectively binds and alters the topology of DNA. The authors suggest diverse regulatory roles for mosquito HMGB family members, for example in vitellogenesis and molting, in addition to innate immunity [63]. This example illustrates the idea that the transcriptome is shaped by multiple environmental factors, and it underscores the importance of performing functional assays to validate any predictions drawn from transcriptomic data.

High-throughput RNAi screens have proved to be a powerful method for identifying candidate flavivirus host and restriction factors in vertebrate and invertebrate systems [37], [64]. Our panel of laboratory and field-derived *A. aegypti* strains with a range of DENV susceptibilities is a valuable tool for functionally characterizing these candidates. We tested the *A. aegypti* orthologs of three candidate DENV host factors identified through an RNAi screen in *Drosophila* cells [37] for ability to modulate resistance to infection by silencing them in three susceptible mosquito strains.

Only the knockdown of a gene encoding an N-acetylglucosamine-6-phosphate deacetylase (N-Gluc, AAEL002430) significantly decreased midgut DENV2-NGC titers, and this effect was only seen in the Rock strain and not in the SIN or PTri strains. This low “success rate” was somewhat unexpected, given the strength of these hits in the initial RNAi screen [37], but it again illustrates the utility of performing functional assays in adult mosquitoes. Interestingly, our microarray analysis revealed that N-Gluc transcripts were significantly and strongly enriched relative to Rock in both PTri and SIN, suggesting the presence of sufficient transcripts for normal functioning even after gene knockdown in these strains.

N-Gluc catalyzes the deacetylation of N-acetylglucosamine-6-phosphate (GlcNAc-6-phosphate) to yield glucosamine-6-phosphate (GlcN-6-P), an intermediate step in the production of uridine diphosphate-GlcNAc (UDP-GlcNAc) [65], [66]. UDP-GlcNAc is a crucial metabolite used in the synthesis of cell walls in bacteria and chitin in yeast and insects, as well as in the GlcNAc moiety of N-linked glycosylation and the glycophosphatidylinositol (GPI) anchor of membrane proteins [65], [67]. Disruption of the N-Gluc gene *nagA* of the cellulose-producing bacterium *Glucoacetobacter xylinus* completely inhibits UDP-GlcNAc synthesis and bacterial growth [65].

N-linked glycosylation of the DENV envelope (E) protein is necessary for virus replication in mammalian cells, but not in mosquito cells or in adult *A. aegypti* [68], [69]. However, there is some evidence that carbohydrate-containing residues such as heparan sulfate, glycosphingolipids, and glycoproteins on the surfaces of both mammalian and mosquito cells bind DENV and may potentially serve as receptors [70], [71]. Carbohydrate-binding agents (CBAs) such as concavalin A, which binds to mannose residues, and wheat germ agglutinin, which binds to GlcNAc, have also been shown to inhibit DENV growth (reviewed in [72]). In our study, one possibility is that N-Gluc depletion via RNAi led to a disruption in UDP-GlcNAc levels, which in turn affected the production or display of receptor glycoproteins or glycosphingolipids on the cell surface, although more experiments are required to substantiate this hypothesis. UDP-GlcNAc is involved in complex metabolic and signaling networks [73], so disruption of the levels of this metabolite has the potential to affect DENV replication via multiple mechanisms.

The susceptibilities to infection with the two dengue virus serotypes (DENV4-WRAIR, DENV2-NGC) differed for some mosquito strains (PTri and SIN) and was similar for others (Orl, Waco, PR, Kitts, BKK). This indicates the existence of mosquito strain- and virus serotype- specific, along with conserved, interactions. We believe that the molecular interactions between the host factors we identified and the virus would likely be quite similar across mosquito and DENV strains, even if they would not affect susceptibility to the same extent. While outside the scope of our study, it will be informative to assess susceptibility by infecting each field-derived mosquito strain with a low passage DENV isolate derived from that particular geographical location of mosquito origin.

In summary, our transcriptomic profiling of a diverse panel of *A. aegypti* strains varying in DENV susceptibility, geographic origin, and passage history has provided valuable insights into patterns of immune gene regulation in natural mosquito populations and has also allowed us to identify new molecular interactions between DENV and *A. aegypti*. Genes whose expression is consistently correlated with DENV susceptibility have the potential to be developed as biomarkers of vector competence, allowing for better targeting of vector control efforts to areas most at risk for dengue epidemics.

## Supporting Information

Figure S1 **Susceptibilities of** ***A. aegypti*** **strains to midgut DENV2 and DENV4 infection.** Midgut virus titers at 7 days post-bloodmeal (dpbm) after infection with (A–D) DENV2-NGC and (E) DENV4-WRAIR. Data are a pool of at least three independent biological replicates, except for DENV4-WRAIR infections and DENV2-NGC infections of PTri, BKK and PR, where two biological replicates were performed. Each data point represents an individual midgut. ****, p≤0.0001; ***, p≤0.001; **, p≤0.01 *, p≤0.05 compared to Rock in Dunn's post-test after Kruskal-Wallis test or in Mann-Whitney test.
(TIF)

Figure S2 **Silencing efficiencies for genes knocked down via RNAi.**
(TIF)

Table S1 **Descriptive statistics for DENV infection assays.** Numbers in parentheses indicate n for each biological replicate.
(DOCX)

Table S2 **Log2-fold values and functional groups of transcripts that were enriched or depleted in each mosquito strain relative to Rock.** Functional group abbreviations: CS, cytoskeletal and structural; CSR, chemosensory reception; DIV, diverse functions; DIG, blood and sugar food digestive; IMM, immunity; MET, metabolism; PROT, proteolysis; RSM, redox, stress and mitochondrion; RTT, replication, transcription, and translation; TRP, transport; UKN, unknown functions. MG, midgut; Car, carcass.
(XLSX)

Table S3 **Numbers of significantly differentially represented transcripts (compared to Rock) in the midgut and carcass of each** ***A. aegypti*** **strain, in total and arranged by functional class.**
(XLSX)

Table S4 **Numerical data for over-represented GO classes in the set of genes that displayed significant differential transcript abundance in the Orl, BKK, PTri, and SIN strains.** Red indicates average log2-fold higher transcript abundance, and green indicates average log2-fold lower transcript abundance for each class. The criteria for over-representation were: at least 3 genes displaying differential transcript abundance per class, z-score >1.96, and p value <0.05. Over-representation analysis was performed with the GO-Elite program (http://www.genmapp.org/go_elite).
(XLSX)

Table S5 **Numerical microarray data for candidate DENV host and restriction factors.** Numbers represent fold-change from Rock strain in log2 scale. Functional group abbreviations: CS, cytoskeletal and structural; CSR, chemosensory reception; DIV, diverse functions; DIG, blood and sugar food digestive; IMM, immunity; MET, metabolism; PROT, proteolysis; RSM, redox, stress and mitochondrion; RTT, replication, transcription, and translation; TRP, transport; UKN, unknown functions. MG, midgut; Car, carcass.
(XLSX)

Table S6 **Primer sequences used for dsRNA synthesis and real-time PCR.**
(XLSX)
