# Supplementary figures and images for: Transcriptomic Profiling of Diverse Aedes aegypti Strains Reveals Increased Basal-level Immune Activation in Dengue Virus-refractory Populations and Identifies Novel Virus-vector Molecular Interactions
Source: PLoS Negl Trop Dis. 2013 Jul 4;7(7):e2295. doi: 10.1371/journal.pntd.0002295 (PMC3701703; doi:10.1371/journal.pntd.0002295)

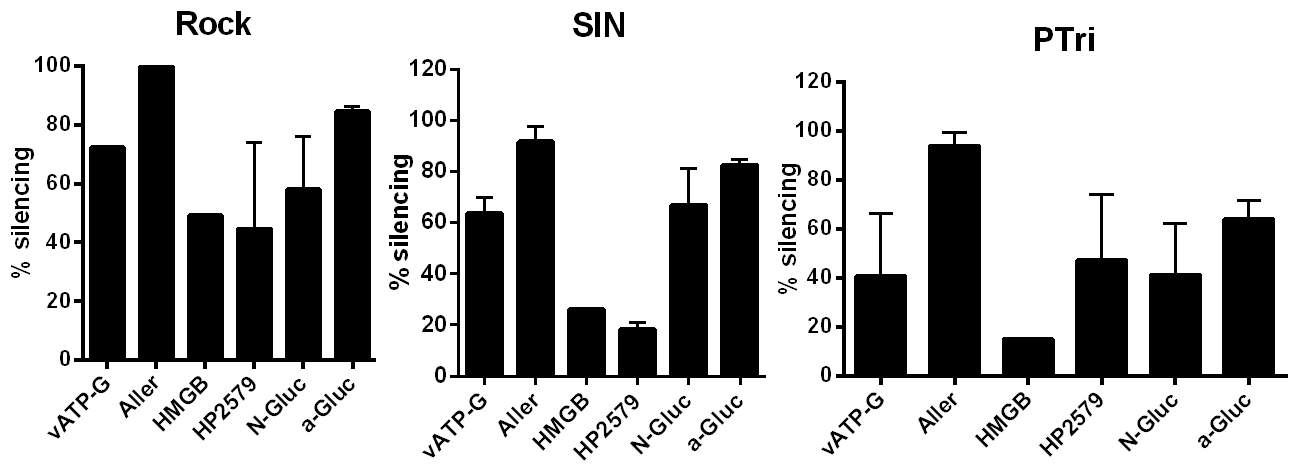

Supplement: Figure S2 — Silencing efficiencies for genes knocked down via RNAi. (TIF) [file pntd.0002295.s002.tif]
