# Supplementary material for: Transcriptomic Profiling of Diverse Aedes aegypti Strains Reveals Increased Basal-level Immune Activation in Dengue Virus-refractory Populations and Identifies Novel Virus-vector Molecular Interactions
Source: PLoS Negl Trop Dis. 2013 Jul 4;7(7):e2295. doi: 10.1371/journal.pntd.0002295 (PMC3701703; doi:10.1371/journal.pntd.0002295)
Supplement: Table S1 — Descriptive statistics for DENV infection assays. Numbers in parentheses indicate n for each biological replicate. (DOCX) [file pntd.0002295.s003.docx]

**Table S1: Descriptive statistics for DENV infections.** Numbers in parentheses indicate n for each biological replicate.

A. DENV2-NGC infection

|  | **Rock** | **Orl** | **Waco** | **PFin** | **Kitts** | **SIN** |
| --- | --- | --- | --- | --- | --- | --- |
| **n** | 83  (30, 23, 30) | 68  (30, 17, 21) | 73  (30, 15, 28) | 48  (23, 14, 11) | 39  (9, 15, 15) | 38  (8, 10, 20) |
| **Mean** | 13077 | 684 | 2078 | 4918 | 5906 | 10305 |
| **Median** | 8500 | 25 | 1050 | 2100 | 1850 | 7500 |
| **SD** | 14174 | 1276 | 2931 | 6804 | 7484 | 10384 |
| **Prevalence (%)** | 90.4 | 58.8 | 93.2 | 66.7 | 92.3 | 84.2 |
| **p-value (Kruskal-Wallis)** | < 0.0001 | | | | | |
| **p-value (Dunn's post test)** | NA | < 0.0001 | < 0.0001 | <0.001 | ns | ns |
| **Relative infection level (%)** | 100 | 0.3 | 12.4 | 24.7 | 21.8 | 88.2 |
|  |  |  |  |  |  |  |
|  | **Rock** | **PTri** | **Rock** | **BKK** | **Rock** | **PR** |
| **n** | 42 (27, 15) | 48 (29, 19) | 46 (28, 18) | 33 (20, 13) | 38 (18, 20) | 50 (30, 20) |
| **Mean** | 17809 | 6914 | 47198 | 5943 | 43142 | 16864 |
| **Median** | 9500 | 5500 | 35000 | 1300 | 30000 | 8250 |
| **SD** | 22333 | 7870 | 37550 | 14743 | 36834 | 22868 |
| **Prevalence (%)** | 95.2 | 93.8 | 100 | 97 | 94.7 | 98 |
| **p-value (Mann Whitney)** | NA | 0.0003 | NA | <0.0001 | NA | <0.0001 |
| **Relative infection level (%)** | 100 | 57.9 | 100 | 3.7 | 100 | 27.5 |

B. DENV4-WRAIR infection

|  | **Rock** | **Orl** | **Waco** | **PR** | **Kitts** | **PTri** | **SIN** | **BKK** |
| --- | --- | --- | --- | --- | --- | --- | --- | --- |
| **n** | 31  (14, 17) | 69  (26, 43) | 46  (27, 19) | 46  (20, 26) | 36  (12, 24) | 29  (24, 5) | 33  (13, 20) | 11  (6, 5) |
| **Mean** | 8328 | 141 | 1288 | 3853 | 6554 | 622.1 | 1591 | 1300 |
| **Median** | 5500 | 10 | 475 | 1700 | 2450 | 450 | 250 | 100 |
| **SD** | 11149 | 301 | 2112 | 4643 | 9073 | 610.3 | 2858 | 2189 |
| **Prevalence (%)** | 96.7 | 55.1 | 95.7 | 95.7 | 91.7 | 93.1 | 69.7 | 63.6 |
| **p-value (Kruskal-Wallis)** | p < 0.0001 | | | | | | | |
| **p-value (Dunn's post test)** | NA | 0.0001 | < 0.01 | ns | ns | < 0.01 | < 0.001 | < 0.001 |
| **Relative infection level (%)** | 100 | 0.3 | 8.8 | 30.8 | 50 | 8.3 | 7.9 | 9.2 |

C. DENV2-NGC vs DENV4-WRAIR infection

|  | **DENV2** | **DENV4** |
| --- | --- | --- |
| **n** | 198 | 31  (14, 17) |
| **Mean** | 23428 | 8328 |
| **Median** | 14000 | 5500 |
| **SD** | 25207 | 11149 |
| **Prevalence** | 95.5 | 96.7 |
| **p-value (Mann Whitney)** | 0.0001 | |

D. DENV2-NGC salivary gland infection

|  | **Rock** | **Orl** | **Kitts** | **SIN** | **BKK** |
| --- | --- | --- | --- | --- | --- |
| **n** | 58 (13, 12, 12, 10, 11) | 60 (7, 9, 1, 11, 16, 11, 5) | 73 (11, 6, 9, 13, 20, 14) | 60 (4, 11, 11, 7, 8, 10, 9) | 41 (9, 6, 11, 8, 2, 5) |
| **Mean** | 7209 | 4194 | 6824 | 3687 | 3167 |
| **Median** | 1800 | 35 | 3000 | 300 | 75 |
| **SD** | 11975 | 9455 | 9071 | 6647 | 5588 |
| **Prevalence** | 87.9 | 50.8 | 67.1 | 67.2 | 53.7 |
| **p-value (Kruskal-Wallis)** | p < 0.01 | | | | |
| **p-value (Dunn's post test)** | NA | < 0.01 | ns | ns | < 0.05 |
| **Relative infection level (%)** | 100 | 1.9 | 166.7 | 16.7 | 4.2 |

E. Pathway RNAi assays

1. Orl

|  | **dsGFP** | **dsMyD88** | **dsImd** | **dsDome** | **dsDcr2** |
| --- | --- | --- | --- | --- | --- |
| **n** | 53 (18, 18, 17) | 41 (16, 10, 15) | 47 (19, 13, 15) | 50 (16, 17, 17) | 47 (16, 11, 20) |
| **Mean** | 5154 | 12062 | 15981 | 11638 | 30184 |
| **Median** | 2250 | 10000 | 10500 | 10000 | 12500 |
| **SD** | 5456 | 13513 | 15931 | 10994 | 34131 |
| **Prevalence (%)** | 92.5 | 100 | 95.7 | 98 | 100 |
| **p-value (Kruskal-Wallis)** | < 0.0001 | | | | |
| **p-value (Dunn's post test)** | NA | < 0.05 | <0.001 | <0.01 | <0.0001 |

2. BKK

|  | **dsGFP** | **dsMyD88** | **dsImd** | **dsDome** | **dsDcr2** |
| --- | --- | --- | --- | --- | --- |
| **n** | 31 (4, 16, 11) | 27 (3, 13, 11) | 34 (7, 19, 8) | 30 (8, 6, 16 | 41 (10, 14, 17) |
| **Mean** | 3436 | 6326 | 12930 | 6480 | 5386 |
| **Median** | 1500 | 5500 | 5000 | 3500 | 1950 |
| **SD** | 3931 | 7105 | 18981 | 6439 | 6451 |
| **Prevalence (%)** | 93.5 | 88.9 | 100 | 93.3 | 92.7 |
| **p-value (Kruskal-Wallis)** | ns | | | | |
| **p-value (Dunn's post test)** | NA | ns | ns | ns | ns |

3. Rock

|  | **dsGFP** | **dsCactus** | **dsCaspar** | **dsPIAS** |
| --- | --- | --- | --- | --- |
| **n** | 49 (18, 18, 13) | 32 (11, 12, 9) | 44 (19, 15, 10) | 60 (21, 16, 23) |
| **Mean** | 10008 | 6231 | 7282 | 5643 |
| **Median** | 9000 | 5500 | 6500 | 5250 |
| **SD** | 5991 | 4963 | 5363 | 3835 |
| **Prevalence (%)** | 98 | 100 | 100 | 100 |
| **p-value (Kruskal-Wallis)** | 0.0006 | | | |
| **p-value (Dunn's post test)** | NA | < 0.05 | ns | < 0.001 |

4. SIN

|  | **dsGFP** | **dsCactus** | **dsCaspar** | **dsPIAS** |
| --- | --- | --- | --- | --- |
| **n** | 31 (9, 11, 11) | 23 (15, 5, 3) | 20 (10, 7, 3) | 22 (9, 7, 6) |
| **Mean** | 25785 | 8807 | 34075 | 20500 |
| **Median** | 15500 | 8000 | 24500 | 14000 |
| **SD** | 28138 | 6015 | 25734 | 19521 |
| **Prevalence (%)** | 100 | 100 | 100 | 100 |
| **p-value (Kruskal-Wallis)** | 0.0001 | | | |
| **p-value (Dunn's post test)** | NA | 0.01 | ns | ns |

F. DENV host factor (1) RNAi assays

1. Rock

|  | **dsGFP** | **dsvATPg** | **dsAller** | **dsHMGB** |
| --- | --- | --- | --- | --- |
| **n** | 83 (26, 27, 30) | 74 (30, 18, 26) | 83 (27, 28, 28) | 66 (26, 16, 24) |
| **Median** | 12000 | 8500 | 9500 | 12000 |
| **Mean** | 21317 | 9197 | 10961 | 17959 |
| **SD** | 25088 | 7143 | 7647 | 19310 |
| **Prevalence (%)** | 100 | 100 | 100 | 98.5 |
| **p-value (Kruskal-Wallis)** | 0.0013 | | | |
| **p-value (Dunn's post test)** | NA | < 0.01 | ns | ns |

2. SIN

|  | **dsGFP** | **dsAller** | **dsHMGB** |
| --- | --- | --- | --- |
| **n** | 54 (8, 38, 7) | 43 (15, 23, 5) | 40 (4, 15, 21) |
| **Median** | 11500 | 14000 | 17250 |
| **Mean** | 11851 | 17031 | 22390 |
| **SD** | 8449 | 13055 | 18795 |
| **Prevalence (%)** | 96.3 | 97.7 | 100 |
| **p-value (Kruskal-Wallis)** | 0.0128 | | |
| **p-value (Dunn's post test)** | NA | ns | < 0.01 |
|  |  |  |  |
|  | **dsGFP** | **dsvATPg** |  |
| **n** | 31 (11, 14, 6) | 29 (13, 9, 7) |  |
| **Median** | 25000 | 16000 |  |
| **Mean** | 36379 | 24762 |  |
| **SD** | 34486 | 23852 |  |
| **Prevalence (%)** | 100 | 100 |  |
| **p-value (Mann Whitney)** | ns | |  |

3. PTri

|  | **dsGFP** | **dsvATPg** | **dsAller** | **dsHMGB** |
| --- | --- | --- | --- | --- |
| **n** | 41 (8, 21, 12) | 45 (13, 15, 17) | 37 (8, 19, 10) | 53 (15, 28, 10) |
| **Median** | 10000 | 6000 | 9500 | 13000 |
| **Mean** | 14489 | 7400 | 14501 | 20244 |
| **SD** | 13484 | 5690 | 15818 | 20140 |
| **Prevalence (%)** | 95.1 | 100 | 100 | 100 |
| **p-value (Kruskal-Wallis)** | 0.0003 | | | |
| **p-value (Dunn's post test)** | NA | < 0.05 | ns | ns |

G. DENV host factor (2) RNAi assays

1. Rock

|  | **dsGFP** | **dsHP2579** | **dsN-Gluc** | **dsa-Gluc** |
| --- | --- | --- | --- | --- |
| **n** | 44 (10, 20, 14) | 44 (14, 14, 16) | 45 (14, 15, 16) | 43 (17, 14, 12) |
| **Median** | 14750 | 10250 | 5500 | 13500 |
| **Mean** | 18933 | 11249 | 8059 | 16512 |
| **SD** | 18377 | 7140 | 7385 | 11567 |
| **Prevalence (%)** | 100 | 100 | 97.8 | 100 |
| **p-value (Kruskal-Wallis)** | < 0.0001 | | | |
| **p-value (Dunn's post test)** | NA | ns | < 0.001 | ns |

2. SIN

|  | **dsGFP** | **dsHP2579** | **dsN-Gluc** | **dsa-Gluc** |
| --- | --- | --- | --- | --- |
| **n** | 31 (11, 14, 6) | 34 (13, 12, 9) | 39 (19, 14, 6) | 17 (9, 3, 5) |
| **Median** | 25000 | 15250 | 16000 | 11000 |
| **Mean** | 36379 | 24146 | 26195 | 18688 |
| **SD** | 34486 | 27102 | 26990 | 27357 |
| **Prevalence (%)** | 100 | 100 | 97.4 | 100 |
| **p-value (Kruskal-Wallis)** | ns | | | |
| **p-value (Dunn's post test)** | NA | ns | ns | ns |

3. PTri

|  | **dsGFP** | **dsHP2579** | **dsN-Gluc** | **dsa-Gluc** |
| --- | --- | --- | --- | --- |
| **n** | 37 (16, 11, 10) | 26 (13, 5, 8) | 35 (20, 8, 7) | 26 (15, 8, 3) |
| **Median** | 15500 | 15750 | 13000 | 9500 |
| **Mean** | 19749 | 27465 | 13921 | 11417 |
| **SD** | 20458 | 36548 | 16383 | 8496 |
| **Prevalence** | 100 | 100 | 97.1 | 96.2 |
| **p-value (Kruskal-Wallis)** | ns | | | |
| **p-value (Dunn's post test)** | NA | ns | ns | ns |
